# Persistent disparities in head and neck cancer survival in the United States

**DOI:** 10.1007/s10552-026-02196-3

**Published:** 2026-06-25

**Authors:** Ella P. Jackert, Swar Vimawala, Liyang Tang, Daniel Kwon, Niels Kokot, Tamara Chambers, Uttam Sinha, Yang Chai, Lihua Liu, Albert Y. Han

**Affiliations:** 1 Keck School of Medicine of USC, 1975 Zonal Avenue, Los Angeles, CA 90033 USA; 2 USC Caruso Department of Otolaryngology Head and Neck Surgery, 1450 San Pablo Street Suite 5800, Los Angeles, CA 90033 USA; 3 Herbert Ostrow School of Dentistry of USC, 925 W 34th Street, Los Angeles, CA 90089 USA; 4 Department of Preventative Medicine of USC, 1845 N Soto Street, Los Angeles, CA 90032 USA

**Keywords:** Head and neck cancer, Disparities, Surgery, Social determinants, Outcomes

## Abstract

**Purpose:**

Head and neck cancer (HNC) is a heterogenous disease with substantial mortality. This study assesses the long-term epidemiologic landscape, demographic disparities, and survival outcomes of HNC.

**Methods:**

Adult patients with primary HNC were identified in the Surveillance, Epidemiology, and End Results (SEER) database by *International Classification of Diseases for Oncology* (ICD-O) codes. The primary outcomes of this study were overall survival (OS) and cancer-specific survival (CSS). Secondary outcomes included primary tumor site distributions and rates of surgery by race and ethnicity. Multivariable Cox regression modeling was utilized to determine predictors of all-cause and HNC-specific mortality.

**Results:**

In this cohort of 117,893 head and neck cancer patients, significant racial and ethnic disparities were observed in tumor site, treatment, and outcomes. Black patients were less likely to receive surgery even when accounting for tumor subsite and stage (OR 0.672, 95% CI 0.641–0.705). Five-year overall survival (OS) and cause-specific survival (CSS) were lower in all minority groups (*p* < 0.001), with Black patients showing the lowest OS (31.3%) and CSS (58.8%). Multivariable analysis confirmed race/ethnicity, low income (HR 1.043, 95% CI 1.018–1.068), and unmarried status (HR 1.327, 95% CI 1.305–1.350) as independent predictors of increased all-cause and HNC-specific mortality.

**Conclusion:**

In this most comprehensive and up-to-date analysis of HNC, we demonstrate profound racial and ethnic disparities in tumor sites, treatments, and survival. This study provides critical evidence to guide health policy, resource allocation, and clinical practice aimed at closing longstanding equity gaps in head and neck cancer care.

## Introduction

Head and neck cancer (HNC) represents a heterogeneous group of highly morbid malignancies that are rising in incidence both in the United States and globally [1–3]. This increase has largely been attributed to human papillomavirus (HPV)-associated oropharyngeal squamous cell carcinoma [4, 5]. In recent years, trends in incidence of HNC have varied especially during the COVID-19 pandemic, contributing to a high disease burden we see today [6]. With recent epidemiologic shifts in the demographic landscape of HNC, racial and ethnic disparities have been revealed that contribute to these evolving incidence rates and survival outcomes [5, 7].

Marked survival disparities exist in HNC, particularly among Black patients, who experience 20–30% poorer survival compared to white patients, which is among the largest racial survival gaps across all cancers [8–11]. This inequity persists compared to other minority groups [12]. In contrast, Hispanic patients may have improved survival outcomes, a phenomenon often referred to as the “Hispanic Health Paradox” [11, 13]. These divergent patterns highlight the complexity of racial and ethnic survival disparities in HNC and underscore the need to identify contributing factors. For example, Black patients have lower prevalence of HPV-associated oropharyngeal squamous cell carcinoma, a HNC subtype with superior prognosis [4, 14, 15]. They are also more likely to experience treatment delays, advanced-stage presentation, have more limited social support, and reduced surgical access [16–19]. When surgery is performed, quality of hospital care is lower, driving survival differences beyond oncologic factors [20]. In addition, Black and Hispanic patients may harbor higher genomic mutation burdens in key genes such as TP53, further compounding socioeconomic and clinical determinants of outcomes [21].

In this context, this study provides the largest and most contemporary database evaluation of racial and ethnic disparities in head and neck cancer in the United States, leveraging three decades of data. We evaluate differences in tumor site, surgical management, and survival across racial and ethnic groups, while accounting for demographic and clinical covariates. By updating survival estimates and identifying key drivers of inequity, our findings hold direct clinical relevance for guiding equitable care delivery and informing future interventions.

## Methods

This study was deemed exempt from institutional review board approval, as it is a secondary analysis of existing data, does not involve intervention or interaction with human subjects, and is fully deidentified in accordance with the deidentification standard defined in Section *§164.514(a)* of the Health Insurance Portability and Accountability Act (HIPAA) Privacy Rule. The study adhered to the Strengthening the Reporting of Observational Studies in Epidemiology (STROBE) guidelines. This study utilized deidentified patient case-level data derived from the 1992 to 2022 Surveillance, Epidemiology, and End Results (SEER) database (National Cancer Institute) [22]. All data were extracted using SEER*Stat version 9.0.41 from August to September 2025.

### Study cohort

The SEER database was queried to identify a study cohort including adult patients 18 years or older with a diagnosis of primary HNC. *International Classification of Diseases for Oncology* (ICD-O) codes for the following sites were included: lip, tongue, floor of mouth, gum and other mouth, salivary gland, tonsil, oropharynx, hypopharynx, nasopharynx, nose/nasal cavity/middle ear, larynx, and other oral cavity and pharynx. The following patients were excluded: those with benign or in situ tumors, those without documented age, and those without documented summary stage.

### Study variables

Patient demographics were extracted, including year of diagnosis, age at diagnosis, sex, race and ethnicity (Non-Hispanic White [White], Non-Hispanic Black [Black], Non-Hispanic Asian or Pacific Islander [Asian], Non-Hispanic American Indian or Alaskan Native [Native American], Hispanic), marital status at time of diagnosis, and inflation-adjusted median annual household income. Marital status was classified as married, unmarried, or unknown. Income was stratified as low (less than $75,000 per year) or high (greater than or equal to $75,000 per year), a cut-point representative of US median annual household income in 2022 [23]. Clinical variables included primary tumor site, summary stage (localized, regional, or distant), primary surgery (yes, no, or unknown), radiation therapy (yes, no/unknown), and chemotherapy (yes, no/unknown). Primary tumor sites, abstracted as ICD-O codes, were grouped as follows to maintain consistency with American Joint Committee on Cancer (AJCC) 8th edition guidelines [24]: oral cavity (lip, tongue, floor of mouth, gum and other mouth), oropharynx, hypopharynx, larynx, salivary gland, nasopharynx, other nasal cavity or middle ear, and other oral cavity and pharynx. Subsite information for the tongue was not available; therefore, both the anterior two-thirds and base of tongue were uniformly categorized under oral cavity tumors.

### Outcomes and statistical analysis

The primary outcomes of interest were 5-year overall survival (OS) and cancer-specific survival (CSS) by racial and ethnic group. Secondary outcomes of interest were primary tumor site specificity and if surgery was done for HNC. Patients with unknown race or ethnicity were excluded from statistical analyses. First, demographics and summary stage were compared across race and ethnicity by Chi-squared tests for categorical variables and two-way *t*-tests for continuous variables. These results were corrected for multiple comparisons via the Bonferroni method [25]. Binary logistic regression was utilized to compare primary tumor site specificity across race and ethnicity with the White cohort as the reference, reported as odds ratios (ORs) with 95% confidence intervals (95% CIs). Odds of receiving surgery were compared across race and ethnicity through multinomial logistic regression to account for confounders of primary tumor site and summary stage. Differences in radiation therapy utilization across racial and ethnic groups were assessed using chi-square tests. Given that SEER records radiation therapy as a binary variable (yes vs. no/unknown) without information on treatment intent, dose, sequencing, or compliance, analyses were limited to unadjusted comparisons, and multivariable modeling was not performed. Survival outcomes by race and ethnicity were determined via Kaplan–Meier curves, with statistical significance set to *p* < 0.05 and assessed by pairwise log-rank tests compared to the White reference cohort. Next, a multivariable Cox regression model was constructed to adjust for demographic (age, sex, race and ethnicity, marital status, and median household income) and clinical (primary tumor site, summary stage, treatments received) covariates as influencers of the hazard of death defined as either all-cause mortality or HNC-specific mortality. Year of diagnosis was also accounted for in the model to mitigate time-dependent variability in outcomes over the last three decades. Lastly, regression analysis was conducted to assess the interaction between race and income which compared patients in the low-income across racial groups. Patients who were alive or lost to follow-up were censored. For cancer-specific survival, patients deceased due to another cause were also censored. All statistical analyses were executed in IBM SPSS Statistics v30.

## Results

### Cohort characteristics

A total of 117,893 patients met inclusion criteria for this study. White patients were older at the time of HNC diagnosis compared to all racial and ethnic minorities at an age of 64.32 $\pm$ 11.92 years (*p* < 0.05) (Table 1). Compared to White patients, of which 55.3% were married at the time of HNC diagnosis, and Asian patients (66.5% married), 61.7% of Black patients were unmarried (*p* < 0.05). Only 36.8% of White patients were low-income. Most Hispanic patients fell within the low household income bracket (50.4%, *p* < 0.05 versus White), while the vast majority of Asian patients fell within the high-income bracket (74.6%, *p* < 0.05 versus White). Black patients were least likely to present at a localized summary stage (27.8%, *p* < 0.05), and White patients were least likely to present at a distant stage (12.1%, *p* < 0.05).

**Table 1:** Cohort characteristics by race and ethnicity

	Mean (SD) or No (%)				
Variable	White	Black	Asian	Native American	Hispanic
Demographics					
Age	64.32 (11.92)	60.77 (11.23)*	61.73 (14.04)*	60.79 (11.39)*	61.69 (12.68)*
Male	64,086 (74.1)	7,427 (75.8)*	6,724 (69.9)*	762 (75.4)	7920 (75.6)*
Marital status at diagnosis					
Married	47,845 (55.3)	3,206 (32.7)*	6,398 (66.5)*	410 (40.6)*	5,384 (51.4)*
Unmarried	34,088 (39.4)	3,039 (61.7)*	2,857 (29.7)*	441 (43.7)	4,493 (42.9)
Median household income					
< $75,000	31,846 (36.8)	3,583 (36.6)	2,442 (25.4)*	293 (29.0)*	5,277 (50.4)*
≥$75,000	54,620 (63.2)	6,209 (63.4)	7,177 (74.6)*	717 (71.0)*	5,190 (49.6)*
Summary stage					
Localized	34,353 (39.7)	2,718 (27.8)*	3,227 (33.5)*	295 (29.2)*	3,605 (34.4)*
Regional	41,660 (48.2)	4,982 (50.9)*	4,706 (48.9)	511 (50.6)	5,093 (48.6)
Distant	10,468 (12.1)	2,092 (21.4)*	1,686 (17.5)*	204 (20.2)*	1,773 (16.9)*
Total (% of 117,893)	86,466 (73.7)	9,792 (8.3)	9,619 (8.2)	1,010 (0.9)	10,467 (8.9)

*p < 0.05 compared to White

*SD* standard deviation

### Site specificity by race and ethnicity

**Fig. 1:**
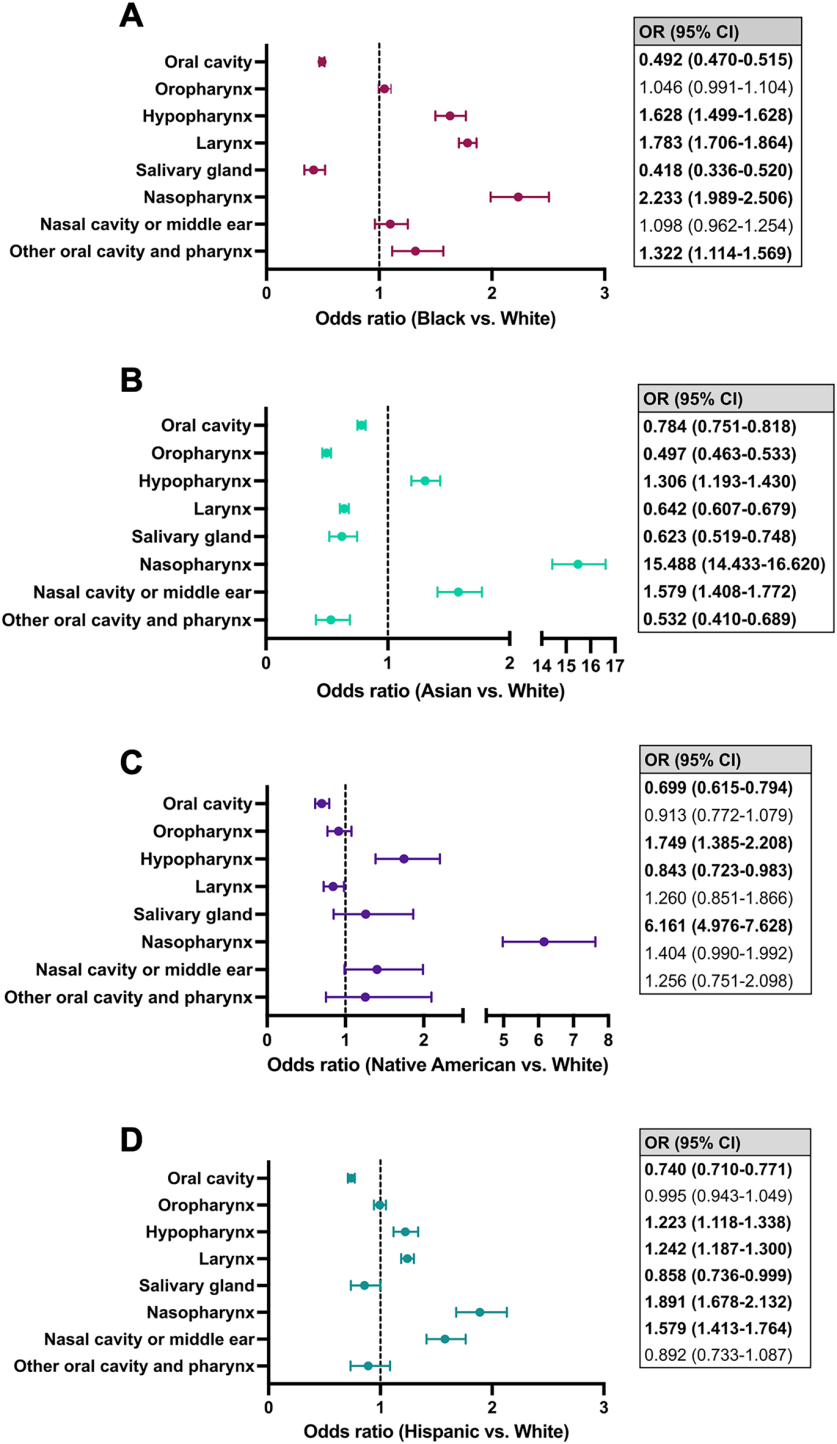
Head and neck cancer site-specificity by race and ethnicity. **A** Site-specificity for Black cohort. **B** Site-specificity for Asian cohort. **C** Site-specificity for Native American cohort. **D** Site-specificity for Hispanic cohort. Odds ratios greater than 1 favor increased odds in minority cohorts. *OR* odds ratio, *95% CI* 95% confidence interval

Though the most common primary site overall, oral cavity tumors were less common among all minority populations studied (ORs: 0.492–0.784; 95% CIs [0.470–0.710 to 0.515–0.818]) compared to White patients (Fig. 1A–D). Black patients had an increased odds of cancers of the hypopharynx (OR 1.628, 95% CI [1.499–1.628]), larynx (OR 1.783, 95% CI [1.706–1.864]), nasopharynx (OR 2.233, 95% CI [1.989–2.506]), and other oral cavity and pharynx (OR 1.322, 95% CI [1.114–1.569]) compared to White patients, but a decreased odds of salivary gland cancers (OR 0.418, 95% CI [0.336–0.520]) (Fig. 1A). Among Asian patients, site-specific differences compared to White patients were largely driven by a significantly increased odds of nasopharynx malignancies (OR 15.488, 95% CI [14.433, 16.620]) (Fig. 1B). Native American patients similarly were at increased odds of nasopharynx cancers (OR 6.161, 95% CI [4.976–7.628]) compared to White patients (Fig. 1C). Hispanic patients were at increased odds of hypopharynx (OR 1.223, 95% CI [1.118–1.338]), larynx (OR 1.242, 95% CI [1.187–1.300]), nasopharynx (OR 1.891, 95% CI [1.678–2.132]), and nose/nasal cavity/middle ear (OR 1.579, 95% CI [1.413–1.764]) malignancies compared to White patients (Fig. 1D).

### Treatment disparities

**Fig. 2:**
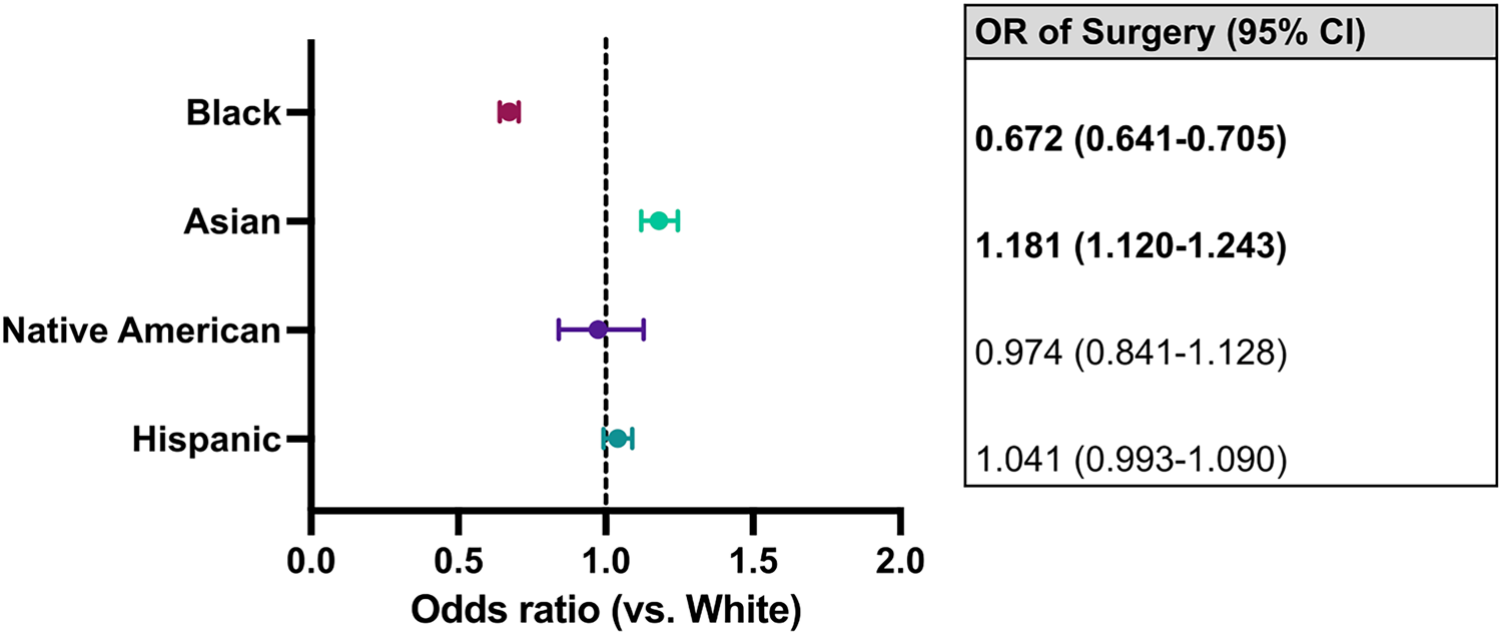
Odds of undergoing surgery by race and ethnicity. Odds ratios greater than 1 favor increased odds of surgery in minority cohorts. *OR* odds ratio, *95% CI* 95% confidence interval

While the majority of the cohort overall received surgery and/or radiation therapy for HNC, there were differences in treatments received across races and ethnicities after accounting for tumor site and summary stage. Black patients were less likely to receive surgery compared to White patients (OR 0.672, 95% CI [0.641–0.705]), while Asian patients were more likely to receive surgery (OR 1.181, 95% CI [1.120–1.243]) (Fig. 2). Radiation therapy was recorded in 64.6% of the overall cohort. Radiation therapy utilization differed across racial and ethnic groups (*p* < 0.001), with the highest recorded receipt among Black (71.3%) and the lowest among White (63.5%) patients. Native American (69.2%) and Asian patients (68.0%) had similar rates of radiation receipt, while receipt was slightly lower in Hispanic patients though not statistically significant (64.1%).

### Overall and cause-specific survival

**Fig. 3:**
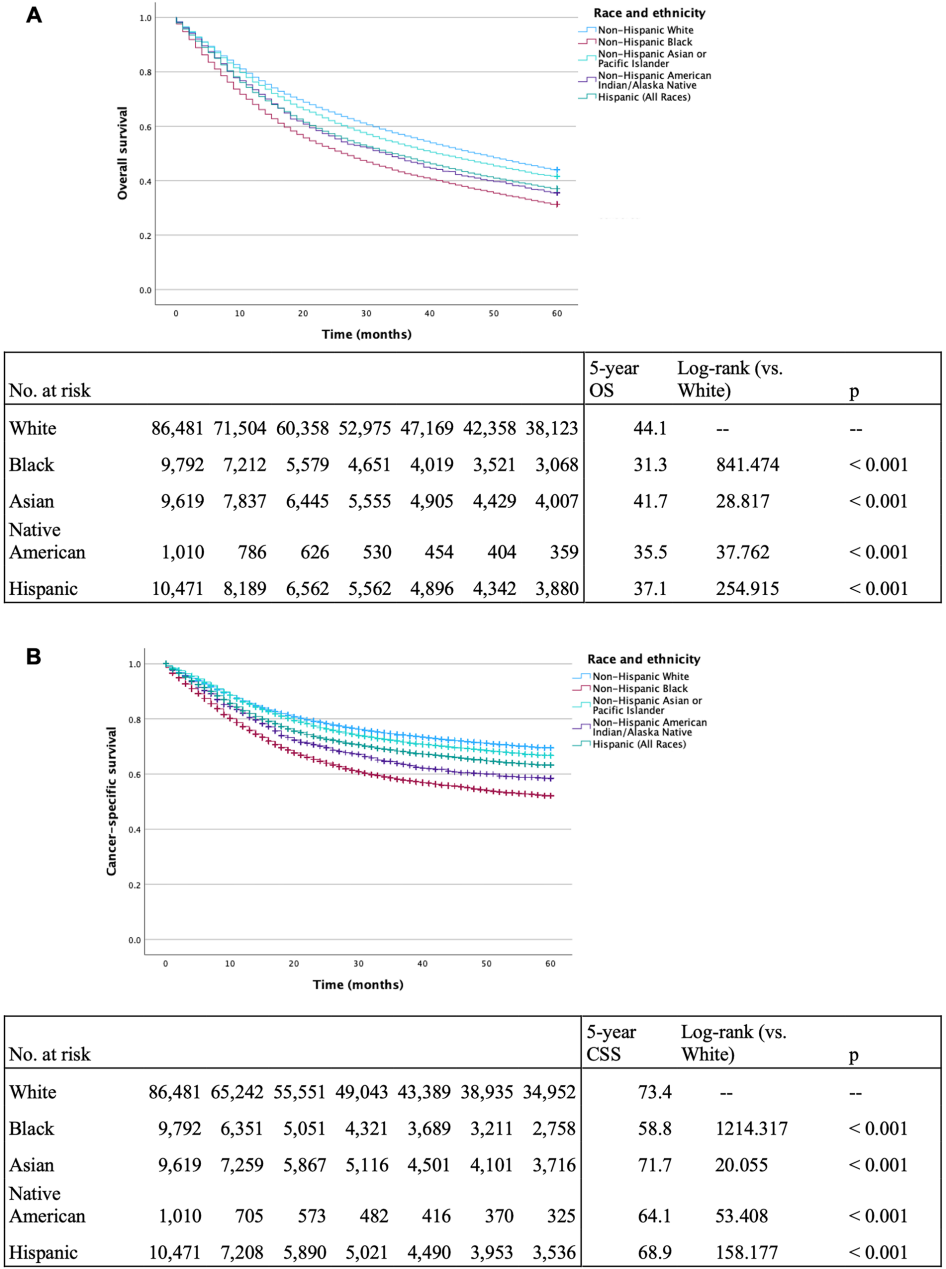
Kaplan–Meier survival curves for 5-year overall survival (**A**) and cancer-specific survival (**B**) by race and ethnicity with corresponding number of patients at risk. Log-rank tests were compared pairwise with White as the reference cohort. *OS:* overall survival, *CSS:* cancer-specific survival

Overall survival (OS) and cause-specific survival (CSS) were assessed through Kaplan–Meier analyses at the 5-year time point. Compared to White patients, all racial and ethnic minorities suffered inferior OS and CSS outcomes (*p* < 0.001) (Fig. 3). Black patients had the lowest OS and CSS survival rates (31.3% and 58.8%, respectively). While the survival difference amongst Black patients was the largest contributor to the overall survival disparities among racial and ethnic minorities, Hispanic patients also experienced significantly decreased OS (37.1%, *X*^2^: 254.915, *p* < 0.001) and CSS (68.9%, *X*^2^: 158.177, *p* < 0.001) which contributed to this disparity.

### Drivers of all-cause and HNC-specific mortality

Finally, multivariable Cox regression analysis identified key demographic and clinical drivers of all-cause and HNC-specific mortality. After accounting for covariates, race and ethnicity remained a significant predictor of both all-cause and HNC-specific mortality (Table 2). Moreover, low household income was significantly associated with HNC-specific mortality (HR 1.043, 95% CI [1.018–1.068]), while higher income somewhat paradoxically predicted all-cause mortality with low household income demonstrating decreased hazard (HR 0.808, 95% CI [0.794, 0.822]). Unmarried status was also significantly associated with both all-cause (HR 1.327, 95% CI [1.305–1.350]) and HNC-specific mortality (HR 1.483, 95% CI [1.447–1.520]). When accounting for the interaction between low income and race and ethnicity, low-income Black, Native American, and Hispanic patients had increased risk of all-cause mortality compared to low-income White patients (HRs 1.130–1.336, 95% CIs [1.083–1.275 to 1.178–1.439], while all low-income minorities had higher HNC-specific mortality compared low-income White patients (HRs 1.130–1.464, 95% CIs [1.045–1.381 to 1.223–1.679] (Table 3).

**Table 2:** Multivariable Cox regression analysis of hazards of all-cause mortality and cancer-specific mortality

	HR (95% CI)	
Variable	All-cause mortality	HNC-specific mortality
Age	**1.026 (1.026–1.027)**	**1.022 (1.021–1.023)**
Female sex (vs. Male)	0.981 (0.963–1.00)	**1.081 (1.045–1.074)**
Race/ethnicity (vs. White)		
Black	**1.370 (1.332–1.408)**	**1.507 (1.452–1.563)**
Asian	**1.083 (1.051–1.115)**	**1.086 (1.040–1.133)**
Native American	**1.227 (1.129–1.333)**	**1.327 (1.186–1.485)**
Hispanic	**1.290 (1.255–1.326)**	**1.199 (1.152–1.248)**
Low household income (vs. high)	**0.808 (0.794–0.822)**	**1.043 (1.018–1.068)**
Unmarried (vs. married)	**1.327 (1.305–1.350)**	**1.483 (1.447–1.520)**
Site	1.000 (0.997–1.003)	**1.015 (1.011–1.091)**
Summary stage (vs. localized)		
Regional	**1.982 (1.941–2.023)**	**3.323 (3.214–3.435)**
Distant	**2.982 (2.904–3.062)**	**6.660 (6.411–6.918)**
Surgery	**0.637 (0.624–0.649)**	**0.587 (0.571–0.603)**

*HR:* hazard ratio, *95% CI:* 95% confidence interval, *HNC:* head and neck cancer; bold indicates statistical significance

**Table 3:** Multivariable Cox regression analysis of hazards of all-cause mortality and cancer-specific mortality associated with the interaction of income and race variables

	HR (95% CI)	
Variable	All-cause mortality	HNC-specific mortality
Race/ethnicity × Low Income (vs. White Low Income)		
Black	**1.336 (1.275–1.400)**	**1.464 (1.381–1.551)**
Asian	0.967 (0.910–1.028)	**1.130 (1.045–1.223)**
Native American	**1.275 (1.090–1.493)**	**1.370 (1.118–1.679)**
Hispanic	**1.130 (1.083–1.178)**	**1.284 (1.215–1.356)**

*HR:* hazard ratio, *95% CI:* 95% confidence interval, *HNC:* head and neck cancer; bold indicates statistical significance

## Discussion

Here, we present the largest analysis of head and neck cancer SEER data to date to highlight pervasive disparities in HNC presentation and outcomes. Our results reveal that social determinants such as marital status and median household income likely intersect with race and ethnicity to produce significant overall and cancer-specific survival disparities of HNC minority patients. Moreover, we clarified differences in primary site-specificity, tumor staging, and treatment disparities. On multivariable analysis accounting for all covariates, race and ethnicity prevailed as a predictor of both all-cause and HNC-specific mortality.

Disparities in HNC tumor subsites are a growing area of study [26]. Some HNC primary site differences have been previously well characterized, such as the high incidence of nasopharyngeal squamous cell carcinoma (NPC) in Asian patients associated with the Epstein-Barr virus (EBV) [27]. Our results are consistent with this prior literature but suggest that NPC is also more common in Black, Native American, and Hispanic patients compared to White patients. Additionally, previous works have highlighted that Black patients with NPC have poorer outcomes compared to White and Asian patients, likely attributed to treatment delays among other socioeconomic factors [28, 29]. Though the Hispanic cohort in this study had increased odds of NPC, there has been no clear survival difference established [30].

Hypopharyngeal and laryngeal primaries were more common among Black and Hispanic patients. Although incidence of laryngeal cancers is decreasing overall in both urban and rural areas, the incidences are disproportionately high in Black patients, with disparities also seen more recently in Hispanic patients [31, 32]. Such disparities have been attributed to income inequality, deficits in education about risks of tobacco and alcohol use, as well as disproportionate occupational exposures [33–35]. Though this study did not specifically assess these exposures, Hispanic patients in this study constituted the largest proportion of the low-income bracket, which thus may contribute to these site differences observed. Targeted outreach and prevention strategies are needed to address the disproportionate burden of hypopharyngeal and laryngeal cancers among Black and Hispanic patients.

Interestingly, odds of oral cavity malignancies were significantly decreased among Black, Asian, Native American, and Hispanic patients compared to White patients, and there were minimal differences in oropharyngeal cancers between cohorts. Though white male patients do make up the majority demographic of oral cavity squamous cell carcinomas, these results warrant further investigation [36]. Of note, the majority of oral cavity cancers were of the tongue subsite. However, there is no differentiation between the anterior two-thirds of the tongue (oral tongue) and base of tongue in ICD-O or other SEER database site codes. The base of tongue is classified as a subsite of oropharyngeal cancer, a category with distinct prognoses leading to specific AJCC staging criteria and treatment guidelines [24]. SEER-derived oral cavity and oropharynx HNC data thus insufficiently differentiates between these key subsites that are known to drive survival differences. Thus, the low odds of oral cavity cancers observed in this study may reflect distributions of base of tongue oropharyngeal primaries. We urge for reconsideration of oral cavity and oropharyngeal HNC classifications with enhanced specificity of tongue subsite in order to more accurately represent the heterogeneous landscape of head and neck cancers and to better assess racial and ethnic differences of these subsites.

Even when accounting for tumor site and summary stage, our results highlighted significant treatment disparities amongst Black patients, who were significantly less likely to receive surgery, which is consistent with prior literature [18, 37]. This phenomenon has been attributed to various compounding socioeconomic factors including insurance status and income [38, 39]. However, the influence of systemic and provider-level factors should not be overlooked, as data show that Black patients are less likely to be offered primary surgery even when clinically appropriate, and those who undergo surgery often experience lower quality postoperative care [20, 37]. Moreover, geographic variables including zip code and distance to advanced cancer care centers likely contributes to decreased access to adequate surgical care, which warrants further study. In contrast, Asian patients were more likely to receive surgery, potentially reflecting the larger proportion of high-income patients in this cohort. Collectively, our results emphasize a need for more equitable treatment recommendations across all patient groups.

Compared to surgical disparities, differences in radiation therapy utilization were more modest, with minority patients, particularly Black patients, demonstrating slightly higher rates of recorded radiation receipt compared to White patients. This likely reflects the central role of radiation therapy in the management of head and neck cancers, particularly in patients with advanced-stage disease who may not be surgical candidates. However, interpretation of these findings is limited by the structure of SEER data, which captures radiation therapy as a binary variable (yes vs. no/unknown) without more comprehensive details on if radiation was definitively received or if patients adhered to guideline-based recommendations. As such, analyses were limited to unadjusted comparisons, and these results should be interpreted cautiously.

Race and ethnicity were significantly associated with both overall and cancer-specific survival in this study. Compared to White patients, Black patients had up to 15% reduced 5-year OS and CSS. These results confirm the well-established trends in survival over the last several decades in which Black HNC patients have poorer prognoses due to a variety of social and clinical factors [8–10]. These prior works have also described the Hispanic health paradox by which patients of Hispanic ethnicity had improved survival compared to White patients [11, 13]. The results of our study of HNC suggest otherwise, as the Hispanic cohort had worse, not better, OS and CSS. Prior works have focused on more limited timeframes, while this study encompasses a broad 30-years of survival data as recent as 2022. Our findings thus suggest a changing landscape in HNC epidemiology, particularly of the Hispanic population. On multivariable analysis, race and ethnicity remained independent predictors of both all-cause and HNC-specific mortality. Other key social determinants including lower household income and unmarried status, which we found to be more common in Hispanic and Black patients, respectively, were also significantly associated with increased mortality risk. Similar social determinants have been described previously, with a particular emphasis on marital status as protective [40, 41]. In our sub-analysis restricted to low-income patients, race and ethnicity remained a significant predictor of both all-cause and HNC-specific mortality, underscoring that race itself functions as an independent prognosticator beyond socioeconomic status. Together, these findings reveal persistent survival gaps for Black patients, an apparent reversal of the Hispanic paradox, and the critical need for enhanced targeted strategies that address both clinical and social drivers of HNC disparities.

This study is highly valuable with its large sample size and long-term data inclusion with high population-level generalizability. However, there are limitations. The use of a retrospective registry analysis subjects this data to residual confounding and potential misclassification biases. The SEER database lacks granular data on factors such as comorbidities, tobacco and alcohol use, and treatment intent, all of which are important prognostic factors. The socioeconomic variables reported are reported at the census-tract or registry level, thus may not fully capture individual-level variation. Coding of radiation in the database groups “no” and “unknown” under the same category, so true differences in receipt of these treatments were unable to be assessed. Moreover, variable treatment adherence may impact results regarding treatment disparities that are not reflected in the database. Staging data is limited to summary stage thus may not capture the updated AJCC staging guidelines by tumor subsite. Additionally, broad categorization of race and ethnicity may fail to fully capture heterogeneity between racial and ethnic subgroups. Insurance status at diagnosis is only captured in SEER beginning in 2007 and was therefore not included in our multivariable models to avoid excluding more than fifteen years of earlier cases.

## Conclusion

This large, contemporary multi-institutional cancer registry study highlights profound and pervasive racial and ethnic disparities in head and neck cancer presentation, treatment, and survival that persist even after accounting for stage, site, and therapy. Our findings emphasize that social determinants, including income and marital status, intersect with race and ethnicity to shape outcomes, underscoring the importance of addressing both clinical and structural drivers of inequity. We also highlight an evolving epidemiology of head and neck cancer with a poorer prognosis observed in all racial and ethnic minorities, including Hispanic patients. Clinically, these results call for intentional strategies to ensure equitable treatment recommendations, improve surgical access, and expand targeted prevention and outreach for disproportionately affected populations.

## Data Availability

The datasets generated and analyzed were derived from the Surveillance, Epidemiology, and End Results database. All analyzed data can be made available from the corresponding author upon reasonable request.

## References

[1] American Cancer Society (2022) Cancer facts & figures 2022. American Cancer Society

[2] Siegel RL, Miller KD, Jemal A (2020) Cancer statistics. CA Cancer J Clin 70(1):7–30. 10.3322/caac.21590 31912902 10.3322/caac.21590

[3] Bray F, Laversanne M, Sung H et al (2024) Global cancer statistics 2022: BLOBOCAN estimates of incidence and mortality worldwide for 36 cancers in 185 countries. CA Cancer J Clin 74(3):229–263. 10.3322/caac.21834 38572751 10.3322/caac.21834

[4] Chaturvedi AK, Engels EA, Pfeiffer RM et al (2011) Human papillomavirus and rising oropharyngeal cancer incidence in the United States. J Clin Oncol 29:4294–4301. 10.1200/JCO.2011.36.4596 21969503 10.1200/JCO.2011.36.4596 PMC3221528

[5] Gormley M, Creaney G, Schache A et al (2022) Reviewing the epidemiology of head and neck cancer: definitions, trends and risk factors. Br Dent J 233:780–786. 10.1038/s41415-022-5166-x 36369568 10.1038/s41415-022-5166-x PMC9652141

[6] Semprini J, Pagedar NA, Boakye EA, Osazuwa-Peters N (2024) Head and neck cancer incidence in the united states before and during the COVID-19 Pandemic. JAMA Otolaryngol Head Neck Surg 150(3):193–200. 10.1001/jamaoto.2023.4322 38206603 10.1001/jamaoto.2023.4322 PMC10784997

[7] Daraei P, Moore CE (2015) Racial disparity among the head and neck cancer population. J Canc Educ 30:546–551. 10.1007/s13187-014-0753-4 10.1007/s13187-014-0753-4 25398667

[8] Karanth SD, Akinyemiji T, Walker CJ et al (2023) The intersectionality between race, ethnicity, and residential-level socioeconomic status in disparities of head and neck cancer outcomes: a SEER study. Cancer Epidemiol Biomarkers Prev 32(4):516–523. 10.1158/1055-9965.EPI-22-1167 36780193 10.1158/1055-9965.EPI-22-1167 PMC10068434

[9] Lara OD, Wang Y, Asare A et al (2020) Pan-cancer clinical and molecular analysis of racial disparities. Cancer 126(4):800–807. 10.1002/cncr.32598 31730714 10.1002/cncr.32598 PMC6992510

[10] Ragin CC, Langevin SM, Marzouk M, Grandis J, Taioli E (2011) Determinants of head and neck cancer survival by race. Head Neck 33(8):1092–1098. 10.1002/hed.21584 20967872 10.1002/hed.21584 PMC3380362

[11] Parasher AK, Abramowitz M, Weed D et al (2014) Ethnicity and clinical outcomes in head and neck cancer: an analysis of the SEER database. J Racial Ethnic Health Disparities 1:267–274. 10.1007/s40615-014-0033-3

[12] Taylor DB, Osazuwa-Peters N, Okafor SI et al (2022) Differential outcomes among survivors of head and neck cancer belonging to racial and ethnic minority groups. JAMA Otolaryngol Head Neck Surg 148(2):119–127. 10.1001/jamaoto.2021.3425 34940784 10.1001/jamaoto.2021.3425 PMC8704166

[13] Marrero-Gonzalez AR, Nanu D, Nguyen SA et al (2025) Disparities in survival of head and neck cancer in the Hispanic population: systematic-review and meta-analysis. Otolaryngol Head Neck Surg 172(4):1177–1191. 10.1002/ohn.1113 39756015 10.1002/ohn.1113 PMC11949715

[14] Osazuwa-Peters N, Massa ST, Christopher KM, Walker RJ, Varvares MA (2016) Race and sex disparities in long-term survival of oral and oropharyngeal cancer in the United States. J cancer Res Clin Oncol 142(2):521–528. 10.1007/s00432-015-2061-8 26507889 10.1007/s00432-015-2061-8 PMC11819284

[15] Faraji F, Rettig EM, Tsai HL (2019) The prevalence of human papillomavirus in oropharyngeal cancer is increasing regardless of sex or race, and the influence of sex and race on survival is modified by human papillomavirus tumor status. Cancer 125(5):761–769. 10.1002/cncr.31841 30521092 10.1002/cncr.31841

[16] Gourin CG, Podolsky RH (2006) Racial disparities in patients with head and neck squamous cell carcinoma. Laryngoscope 16(7):1093–1106. 10.1097/01.mlg.0000224939.61503.83 10.1097/01.mlg.0000224939.61503.83 16826042

[17] Ioerger P, Mills K, Wagoner SF (2024) Inequities associated with advanced stage at presentation of head and neck cancer: a systematic review. JAMA Otolaryngol Head Neck Surg 150(8):727–740. 10.1001/jamaoto.2024.1180 38935363 10.1001/jamaoto.2024.1180

[18] Nocon CC, Ajmani G, Bhayani MK (2019) A contemporary analysis of racial disparities in recommended and received treatment for head and neck cancer. Cancer 126(2):381–389. 10.1002/cncr.32342 31580491 10.1002/cncr.32342

[19] Naghavi AO, Echevarria MI, Strom TJ et al (2016) Treatment delays, race, and outcomes in head and neck cancer. Cancer Epidemiol 45:18–25. 10.1016/j.canep.2016.09.005 27664388 10.1016/j.canep.2016.09.005

[20] Jassal JS, Cramer JD (2021) Explaining racial disparities in surgically treated head and neck cancer. Laryngoscope 131(5):1053–1059. 10.1002/lary.29197 33107610 10.1002/lary.29197

[21] Wu ES, Park JY, Zeitouni JA et al (2016) Effect of actionable somatic mutations on racial/ethnic disparities in head and neck cancer prognosis. Head Neck 38(8):1234–1241. 10.1002/hed.24420 27028310 10.1002/hed.24420

[22] National Cancer Institute. Surveillance, Epidemiology, and End Results (SEER) program. http://www.seer.cancer.gov. Accessed 10 Aug 2025.

[23] Guzman G, Kollar M (2023) Income in the United States: 2022. US Census Bureau. Accessed 15 Sep 2025.

[24] Amin MB, Edge SB, Greene FL et al (2017) AJCC Cancer Staging Manual, 8th edn. Springer

[25] Armstrong RA (2014) When to use the Bonferroni correction. Ophthalmic Physiol Opt 34(5):502–508. 10.1111/opo.12131 24697967 10.1111/opo.12131

[26] Mazul AL, Chidambaram S, Zevallos JP, Massa ST (2022) Disparities in head and neck cancer incidence and trends by race/ethnicity and sex. Head Neck 45(1):75–84. 10.1002/hed.27209 36200577 10.1002/hed.27209 PMC9742317

[27] Wang Y, Zhang Y, Ma S (2013) Racial differences in nasopharyngeal carcinoma in the United States. Cancer Epidemiol 37(6):793–802. 10.1016/j.canep.2013.08.008 24035238 10.1016/j.canep.2013.08.008 PMC3851929

[28] Zhou L, Shen N, Li G, Ding J, Liu D, Huang X (2019) The racial disparity of nasopharyngeal carcinoma based on the database analysis. Am J Otolaryngol 40(6):102288. 10.1016/j.amjoto.2019.102288 31526630 10.1016/j.amjoto.2019.102288

[29] Dee EC, Wang S, Ho FD et al (2024) Nasopharynx cancer in the United States: racial and ethnic disparities in stage at presentation. Laryngoscope 135(3):1113–1119. 10.1002/lary.31907 39548864 10.1002/lary.31907 PMC12333929

[30] Patel VJ, Chen NW, Resto VA (2016) Racial and ethnic disparities in nasopharyngeal cancer survival in the united states: a SEER study. Otolaryngol-Head Neck Surg 156(1):122–131. 10.1177/0194599816672625 27703094 10.1177/0194599816672625

[31] Pagedar NA, Khal AR, Tasche KK et al (2019) Incidence trends for upper aerodigestive tract cancers in rural United States counties. Head Neck 41(8):2619–2624. 10.1002/hed.25736 30843640 10.1002/hed.25736 PMC7133543

[32] Mousavi S, Ilaghi M, Aslani A et al (2024) Laryngeal cancer incidence trends in the United States over 2000–2020: a population-based analysis. Arch Public Health 82:106. 10.1186/s13690-024-01333-1 38987838 10.1186/s13690-024-01333-1 PMC11234729

[33] Tang JA, Lango MN (2019) Diverging incidence trends for larynx and tonsil cancer in low socioeconomic regions of the US. Oral Oncol 91:65–68. 10.1016/j.oraloncology.2019.02.024 30926064 10.1016/j.oraloncology.2019.02.024

[34] Nguyen-Grozavu FT, Pierce JP, Sakuma KK et al (2020) Widening disparities in cigarette smoking by race/ethnicity across education level in the United States. Prev Med 139:106220. 10.1016/j.ypmed.2020.106220 32693179 10.1016/j.ypmed.2020.106220 PMC7494609

[35] Menvielle G, Daniele L, Goldbert P, Leclerc A (2004) Smoking, alcohol drinking, occupational exposures and social inequalities in hypopharyngeal and laryngeal cancer. Int J Epidemiol 33(4):799–806. 10.1093/ije/dyh090 15155704 10.1093/ije/dyh090

[36] Funk GF, Karnell LH, Robinson RA, Zhen WK, Trask DK, Hoffman HT (2002) Presentation, treatment, and outcome of oral cavity cancer: a national cancer data base report. Head Neck 24(2):165–180. 10.1002/hed.10004 11891947 10.1002/hed.10004

[37] Whitehead RA, Patel EA, Liu JC, Bhayani MK (2024) Racial disparities in head and neck cancer: it’s not just about access. Otolaryngol Head Neck Surg 170(4):1032–1044. 10.1002/ohn.653 38258967 10.1002/ohn.653

[38] Subramian S, Chen A (2013) treatment patterns and survival among low-income medicaid patients with head and neck cancer. JAMA Otolaryngol Head Neck Surg 139(5):489–495. 10.1001/jamaoto.2013.2549 23598992 10.1001/jamaoto.2013.2549

[39] Nallani R, Subramanian TL, Ferguson-Square KM et al (2022) A Systematic review of head and neck cancer health disparities: a call for innovative research. Otolaryngol Head Neck Surg 166(6):1238–1248. 10.1177/01945998221077197 35133913 10.1177/01945998221077197

[40] Osazuwa-Peters N, Adjei Boakye E, Chen BY, Tobo BB, Varvares MA (2018) Association between head and neck squamous cell carcinoma survival, smoking at diagnosis, and marital status. JAMA Otolaryngol Head Neck Surg 144(1):43–50. 10.1001/jamaoto.2017.1880 29121146 10.1001/jamaoto.2017.1880 PMC5833596

[41] Inverso G, Mahal B, Aizer AA, Donoff RB, Chau NG, Haddad RI (2015) Marital status and head and neck cancer outcomes. Cancer 121(8):1273–1278. 10.1002/cncr.29171 25524565 10.1002/cncr.29171

